# Precision Estimation of Crop Coefficient for Maize Cultivation Using High-Resolution Satellite Imagery to Enhance Evapotranspiration Assessment in Agriculture

**DOI:** 10.3390/plants13091212

**Published:** 2024-04-27

**Authors:** Attila Nagy, Nikolett Éva Kiss, Erika Buday-Bódi, Tamás Magyar, Francesco Cavazza, Salvatore Luca Gentile, Haidi Abdullah, János Tamás, Zsolt Zoltán Fehér

**Affiliations:** 1 Faculty of Agricultural and Food Sciences and Environmental Management, Institute of Water and Environmental Management, University of Debrecen, H-4032 Debrecen, Hungary; attilanagy@agr.unideb.hu (A.N.); bodi.erika@agr.unideb.hu (E.B.-B.); magyar.tamas@agr.unideb.hu (T.M.); tamas@agr.unideb.hu (J.T.); feher.zsolt@agr.unideb.hu (Z.Z.F.); 2 Consorzio di Bonifica Canale Emiliano Romagnolo, Via E. Masi 8, 40137 Bologna, Italy; cavazza@consorziocer.it (F.C.); gentile@consorziocer.it (S.L.G.); 3 Faculty of Geo-Information Science and Earth Observation (ITC), University of Twente, Drienerlolaan 5, P.O. Box 217, 7500 AE Enschede, The Netherlands; h.j.abdullah-1@utwente.nl

**Keywords:** Sentinel-2, vegetation index-based K_c_, vegetation index-based crop evapotranspiration, maize water demand

## Abstract

The estimation of crop evapotranspiration (ETc) is crucial for irrigation water management, especially in arid regions. This can be particularly relevant in the Po Valley (Italy), where arable lands suffer from drought damages on an annual basis, causing drastic crop yield losses. This study presents a novel approach for vegetation-based estimation of crop evapotranspiration (ETc) for maize. Three years of high-resolution multispectral satellite (Sentinel-2)-based Normalized Difference Vegetation Index (NDVI), Normalized Difference Water Index (NDWI), Normalized Difference Red Edge Index (NDRE), and Leaf Area Index (LAI) time series data were used to derive crop coefficients of maize in nine plots at the Acqua Campus experimental farm of Irrigation Consortium for the Emilia Romagna Canal (CER), Italy. Since certain vegetation indices (VIs) (such as NDVI) have an exponential nature compared to the other indices, both linear and power regression models were evaluated to estimate the crop coefficient (K_c_). In the context of linear regression, the correlations between Food and Agriculture Organization (FAO)-based K_c_ and NDWI, NDRE, NDVI, and LAI-based K_c_ were 0.833, 0.870, 0.886, and 0.771, respectively. Strong correlation values in the case of power regression (NDWI: 0.876, NDRE: 0.872, NDVI: 0.888, LAI: 0.746) indicated an alternative approach to provide crop coefficients for the vegetation period. The VI-based ETc values were calculated using reference evapotranspiration (ET_0_) and VI-based K_c_. The weather station data of CER were used to calculate ET_0_ based on Penman-Monteith estimation. Out of the Vis, NDWI and NDVI-based ETc performed the best both in the cases of linear (NDWI RMSE: 0.43 ± 0.12; NDVI RMSE: 0.43 ± 0.095) and power (NDWI RMSE: 0.44 ± 0.116; NDVI RMSE: 0.44 ± 0.103) approaches. The findings affirm the efficacy of the developed methodology in accurately assessing the evapotranspiration rate. Consequently, it offers a more refined temporal estimation of water requirements for maize cultivation in the region.

## 1. Introduction

The ongoing trends in climate change are exerting profound implications on various facets of human society, particularly impacting agriculture, and the global food supply [1]. Improving agricultural water use efficiency and crop water productivity are the pillars of attaining higher agricultural production. Consequently, the optimal consumption of limited water resources is becoming of primary importance for the public sector, especially in South Europe [2]. This is particularly true in the Mediterranean region, where multiple stakeholders (agriculture, household, industry) compete for increasingly scarce water resources [3]. This is especially true in agricultural regions (e.g., northern Italy, Emilia Romagna), with water-intensive (maize, tomato) agricultural production. Changes in temperature, rainfall patterns, atmospheric carbon dioxide (CO_2_), and the frequency and intensity of extreme weather events all have a substantial influence on agricultural yields, leading to rising food insecurity [4]. Proper water management in agriculture is a key factor in agricultural production.

Due to the high spatiotemporal and temporal variability of meteorological parameters and vegetation as well as other environmental factors, such as soil conditions or the depth of the water table, planning optimal distribution of irrigation water is a very complex task [5]. Such highly heterogeneous environments can be surveyed as detailed as possible resulting in a large variety of data. Meanwhile, data-intensive modern artificial intelligence, pattern recognition, and neural networks may contribute to more efficient planning and assessment of irrigation water distribution [6]. On the other hand, the proper estimation of evapotranspiration (ETc) is a key parameter in the estimation of crop water demand for irrigation planning, design, and efficient irrigation water management of irrigation schemes [7,8]. One of the most commonly used methods for ETc estimation is the crop coefficient approach, which can be calculated based on reference evapotranspiration (ET_0_) and crop coefficient (K_c_) [9]. The K_c_ changes throughout the growing season and reflects the proportion of the ground cover of the vegetation [10]. Several approaches can be used to measure and/or estimate crop evapotranspiration (ETc), such as eddy covariance, soil water balance approach, Bowen ratio energy balance system, pan evaporation, and weighting lysimeters [11]. However, these methods as well as ETc-based soil water balance models are expensive, hard to automate, and only provide point-based measurements. Hence, the application of these models encounters challenges when extended to expansive regions due to the spatial and temporal heterogeneity of input data. This is particularly notable as local conditions, including soil hydraulic parameters, interactions with groundwater, and variations in plant growth, pose intricate considerations that are not easily accounted for [12].

To tackle such challenges, remote sensing (RS) offers low-cost labor-saving, and time-saving approaches for assessing the spatial and temporal patterns of vegetation at different scales [13,14]. Satellite-based global estimates of evapotranspiration are available like MOD16 (MODIS Global Evapotranspiration) with 1 km spatial resolution including both ET_0_, ETc estimates [15] or ECOsystem Spaceborne Thermal Radiometer Experiment on the Space Station (ECOSTRESS) with 70 m spatial resolution [16]. The combination of different satellite data sources enables state-of-the-art and very sophisticated assessment methods. Traditionally, optical and thermal cameras (i.e., Surface Energy Balance Algorithm for Land (SEBAL) [17], Two Source Energy Balance (TSEB) [18], Surface Energy Balance System (SEBS) [19], Mapping Evapotranspiration at High Resolution with Internalized Calibration (METRIC) [20], and the Simplified Surface Energy Balance (SSEB) [21] and others) that are coupled with synthetic aperture radars can provide a variety of near real-time snapshots of the environment, which can contribute to modern agricultural methods [22,23,24]. Earth observation (EO) approaches offer accurate data for precision agriculture applications and end-users (i.e., farmers, landowners, and decision-makers). High-resolution multispectral images may be used to schedule irrigation depending on crop demands in near real-time [25,26]. Despite substantial research on ET crop estimates for water management using EO data, the poor geographical and temporal resolution of the sensors has hindered their usefulness and technical transfer [27]. However, precision irrigation requires high-resolution estimates of crop evapotranspiration and irrigation water requirements at the field level. There are several other hydrological models which use ETc as an input parameter as well [28]. Using biomass-based daily estimation of K_c_ over the vegetation period fosters a more reliable estimation of evapotranspiration contributing to the calculation of a more accurate water balance [29,30].

In the crop coefficient approach, K_c_ is typically taken from the Food and Agricultural Organization, FAO [31], which does not capture the spatial and temporal variability in estimating crop evapotranspiration. However, proper detection of crop growth affects the estimates of evapotranspiration fluxes and irrigation needs. Nowadays, high-resolution satellite datasets (10 m of spatial resolution) such as Sentinel-2 are easy and free to access online, therefore the spatial and temporal variability in the K_c_ can be easily derived from VIs considering actual biomass, crop growth conditions, and stages [32]. The Sentinel-2 A/B was used in this study, with five days of returning time. Free satellite imagery, high-resolution, innovative spectrum capabilities, a 290 km swath width, and frequent return durations are boosting operational and commercial uses of EO data for precision agriculture applications and research initiatives [33,34]. The recent Sentinel-2 mission from the European Space Agency (ESA), as part of the Copernicus program [35], has significantly enhanced regular monitoring of agricultural parameters, like Normalized Difference Vegetation Index (NDVI), Normalized Difference Red Edge Index (NDRE), Normalized Difference Water Index (NDWI) and Leaf Area Index (LAI) [36,37,38]. NDVI is a widely used VI and could provide important information for crop modeling research [39]. NDVI time series are applied to estimate crop growth under different climatic conditions [40]. The NDVI data has been used to monitor crop conditions in irrigation management [41] and estimate crop yield under different agroecological conditions [42,43,44]. The spatial and temporal variability in K_c_ can also be easily derived from NDVI because actual crop growth conditions and stages influence the temporal changes in NDVI values [32]. The NDRE uses the reflectance from red edge bands to estimate plant health [45]. The NDRE responds to plant health, biomass, and photosynthetic activity. It is, therefore, appropriate for plant health and productivity monitoring [46,47]. The NDWI is a commonly used VI to determine the difference between water and vegetation cover, using the differences in the visible and near-infrared wavelength ranges [48]. It is used to delineate, measure, and track the size and the spatial extent of change in water bodies [49] as well as to assess the effect of soil moisture or drought stress on plants [50]. The monitoring of LAI distribution and variation is essential for tracking and detecting vegetation vitality and growth [51]. LAI value may also be determined from RS data by empirical relationships between canopy reflectance and VI regardless of time or costs [52].

This research evaluates the use of RS-based indices in ETc calculation for the region of Emilia Romagna, Italy. There is an IT Irrigation Advisory Service for Farm Water Management services (IRRIFRAME) which utilizes a water balance model that aims at supporting crop irrigation management on a field scale and results in seasonal water savings of about 120 million m^3^ in Italy [53]. The IRRIFRAME provides evapotranspiration data under standard conditions [54], calculating reference evapotranspiration data (ET_0_) by the Hargreaves formula with a 6.25 km^2^ geographical grid. This study aimed to develop an advanced RS-based crop coefficient (K_c_) and crop evapotranspiration (ETc) estimation method to support the preciseness of the calculation of the potential maize water consumption with 10 m spatial resolution. Sentinel-2-derived remotely sensed indices (i.e., NDVI, NDWI, NDRE, and LAI) were integrated with climatic data to define (i) the lengths of the phenological phases, (ii) biomass-based crop coefficient and (iii) to calculate and evaluate more accurate RS-based crop evapotranspiration. Due to the 5-day revisit time of Sentinel-2 satellites, the results contribute to the timely calculation of crop evapotranspiration. Results could also contribute to further improvement in spatial resolution (10 m) of the water balance calculations at maize fields.

## 2. Materials and Methods

### 2.1. Study Site

Field trials were carried out by the Irrigation Consortium for the Emilia Romagna Canal (CER) at the experimental farm Acqua Campus, covering 12.5 hectares of area that can be divided into approximately 25 fields. The fields are located in the plain of the Po River, in the province of Bologna, near the village of Mezzolara di Budrio (44°34′ N, 11°32′ E). Various crops are cultivated on the farm (both perennial and annual). Crop rotation in the experimental fields is characterized by annual crops, such as winter wheat, soya, maize, onion, and processing tomato.

The farm’s soil is typical of the plain of the Po River and has a high content of silt, clay, and fine sand. The soil in the area can be described as clay/clayey loam. It belongs to the Italian soil group “Suoli SECCHIA franco argillosi”, which can be classified as “Oxyaquic Haplustepts” (fine loamy, mixed, superactive, and mesic according to Soil Taxonomy). These soils are calcareous and moderately alkaline; they have a texture of clay loam in the superficial layers and loam deeper in the profile. Soil layers are affected by multiple floods of the nearby Idice River. The soil within the farm is heterogeneous, and its hydraulic characteristics vary from field to field and layer to layer, with a slight gradient from east to west. The mean values for the soil parameters are specified in Table 1. An extended, shallow groundwater table usually is present at a depth ranging from −0.6 to −1.8 m. During winter and at the beginning of the growing season, the capillary rise could be significant in terms of replenishing the evapotranspiration of crops.

For the experimental farm, the water is supplied from the CER canal system, and its quality is regularly checked during the irrigation season [55]. CER’s irrigation water characteristics (long-term mean) are freely available online [56].

The farm has a fully operational weather station equipped with a rain gauge, anemometer, phreatimeter, and pan evaporimeter.

**Figure 1:**
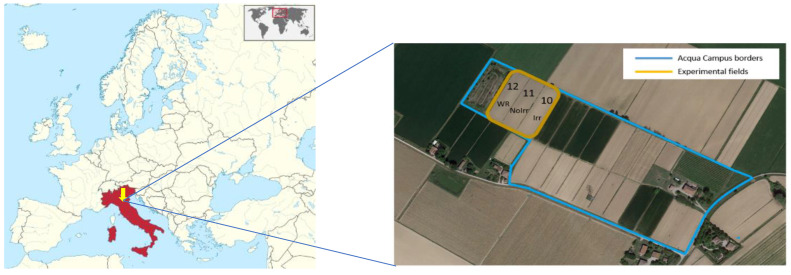
Experimental fields on the Acqua Campus farm.

The climate of the site can be defined as sub-humid, with a mean annual temperature of 13.7 °C and a mean annual rainfall of 771 mm. The year 2018 of the field trials was characterized by severe drought. According to the estimates based on CER analyses, 2018 was the second harshest year in the study area, with a cumulative precipitation mostly below 200 mm in all areas which represents a deviation of 50% from the reference climate (period 1961–2018). These conditions contributed significantly to maize production and yield values. In 2019 and 2020, the temperature was above the regional average with occasional heatwaves; however, the precipitation exceeded 200 mm in the vegetation period of maize. For this study, three experimental fields were cultivated with maize on the Acqua Campus farm as shown in Figure 1. According to the CER data, the crop yield varied between 16.5 and 16.7 t/ha in 2018, 13.8 and 16.2 t/ha in 2019, and 18.8 and 19.9 t/ha in 2020, respectively.

### 2.2. Study Framework and Data

In this study, data from three maize fields in three years (2018, 2019, 2020) were used to set an advanced RS-based crop evapotranspiration estimation method to support a more accurate calculation of the water consumption of maize. The daily meteorological data (minimum and maximum air temperature, relative humidity, radiation, and wind speed) were used to estimate ET_0_ using the Penman-Monteith method. The crop coefficient was defined by developing linear and power regression equations between the NDVI, NDWI, NDRE LAI, and FAO K_c_ for the maize crop. Then VI-based K_c_ was used to calculate ET_c_, and the performances of the index-based crop evapotranspiration were compared to ET_c_ derived by FAO K_c_.

The analysis was conducted using a diverse set of datasets, incorporating Sentinel-2 satellite-derived NDVI, NDWI, NDRE, and LAI datasets, each possessing a high spatial resolution of 10 m. The LAI was calculated from the Sentinel-2 data using the methodology developed in the S2ToolBox within SNAP 5.0. S2ToolBox is an essential component of the SNAP 5.0 software and was specifically developed for LAI retrieval. The methodology uses the approach of an Artificial Neural Network (ANN) to derive these parameters from instantaneous observations of Sentinel-2. By utilizing a pre-trained neural network, the necessary biophysical variables are rapidly retrieved for each pixel within the selected Sentinel-2 image. The training database is generated using a Radiative Transfer Model (RTM) [57].

These datasets were acquired through the VULTUS API (https://api.vultus.se/graphql (accessed on 15 February 2021)). The temporal resolution of the indices was set at five days, aligning with the satellite’s revisit time. A comprehensive dataset, comprising 28 Vegetation Indices (VI) images and associated data, was annually collected, spanning from sowing to harvest. Only data free from cloud cover were considered for this study. This resulted in a final dataset of 996 observations. To ensure temporal coherence in the data, a Piecewise Cubic Hermite Interpolating Polynomial (PCHIP) approach was employed, resulting in a smoothed daily curve for indices throughout the vegetation period. While Acqua Campus of CER provided the weather data (daily minimum and maximum temperature, wind speed, relative humidity, and solar radiation) using an in situ weather station, as well as the Biologische Bundesanstalt, Bundessortenamt and CHemical industry (BBCH) [58] and LAI datasets (local observations). The weather data were used for calculating ET_0_, whilst BBCH and LAI were used to define the lengths of the phenological stages. Crop phenophases were assessed qualitatively by biweekly survey and sampling of maize plants in the field. The BBCH scale is used to identify the lengths of phenological development stages of plants [59]. LAI measurement was carried out to assess the length of phenological stages through direct methods, which involves the removal of plants in the field on certain test areas and measuring their area in the laboratory. More details for LAI measurements are described by Nagy et al. [60].

### 2.3. VI-Based ET_c_ Calculations

The crop evapotranspiration ET_c_ was calculated using the following equation (ET_c_ = ET_0_ × K_c_). ET_0_ was defined by temperature, relative air humidity, solar radiation, wind speed, and air pressure according to the FAO Penman-Monteith equation [10] (Equation (1)).

(1) $${ET}_{0}=\frac{0.408∆\left(R_{n}-G\right)+\gamma \frac{900}{T+273}u_{2}\left(e_{s}-e_{a}\right)}{∆+\gamma \left(1+0.34u_{2}\right)}$$

where:

- ET_0_: reference crop evapotranspiration (mm day^−1^),
- R_n_: net radiation at the crop surface (MJ m^−2^ day^−1^),
- G: soil heat flux density (MJ m^−2^ day^−1^),
- T: mean daily air temperature at 2 m height (°C),
- u_2_: wind speed at 2 m height (m s^−1^),
- e_s_: saturation vapor pressure (kPa),
- e_a_: actual vapor pressure (kPa),
- e_s_ − e_a_: saturation vapor pressure deficit (kPa),
- Δ: slope vapor pressure curve (kPa °C^−1^),
- γ: psychrometric constant (kPa °C^−1^).

Based on the BBCH and LAI, the lengths of the phenological stages were calculated for the three assessed years (Table 2). In general, the sowing time was in late March or early April, the harvesting was in September. The initial stage starts on the day of sowing and continues until about 10% of the soil cover is reached. The early root growth of maize makes it particularly sensitive to drought stress. In the early stages, evaporation is mainly in the form of soil evaporation and the leaf area is small. The mid-season is characterized by rapid growth of maize, from 10% soil cover to actual full soil cover. As the plant grows and covers a larger area of soil, transpiration gradually takes over as the primary mechanism, while evaporation becomes more limited, increasing water use. Water demand is highest in the mid-season stage due to high transpiration, increased biomass production, and grain formation, and K_c_ reaches its maximum value. In the late season stage, transpiration-related water demand may decrease, but at this stage, drought stress can still have a significant negative impact on productivity [61,62,63].

Since the determination method of K_c_ presented in the FAO-56 document shows a trapezoid development, and the crop coefficient is defined as a constant throughout the initial and mid-stage, it does not reflect the natural continuous process of canopy development [32]. This approach is referred to as “original” in this study. Therefore, in this study, the trapezoid character of the FAO K_c_ was smoothed to have continuously varying characteristics by the application of two technical approaches curve with continuously varying values to be closer to natural crop development. One of the approaches was to cut 10 days around the breakpoints and the spline algorithm was implemented to estimate missing values. The solution is called “curved” in this study. The other approach was the so-called “MidPoint”, where only the middle stage plateau was determined by its middle point and the length of the plateau phase.

To analyze the relationship between the FAO-56-based (“original”, “MidPoint”, “curved”) K_c_ values and the four Sentinel-2 derived indices (NDVI, NDRE, NDWI, and LAI), Pearson Product-Moment correlation was applied. Based on the results, FAO-56-based K_c_ was selected, which had the highest correlation with the Vis. Then linear and power regression models were developed based on VI data. Since certain Vis (such as NDVI) have proved to be an exponential nature compared to the other indices, both linear and power regression models were evaluated [61,64,65,66]. The use of standard linear or power regression models with standard estimation techniques is subject to several conditions regarding the explanatory (x) (which were the Vis) and output (y) variables (which were the K_c_ values) and their relationship.

Using the Vis-based estimated K_c_ and FAO-56 K_c_, standard ET_c_ was calculated for all parcels for each year, resulting in biomass-derived evapotranspiration for the vegetation period of the maize fields. To evaluate the VI-based estimation ET_c_ models, the following statistical indicators were selected: adjusted R^2^ (R^2^_adj_) (Equation (2)), Root Mean Square Error (RMSE) (Equation (3)), Normalized Root Mean Square Error (NRMSE) (Equation (4)), Mean Bias Errors (MBE) (Equation (5)) and Mean Absolute Error (MAE) (Equation (6)). RMSE is the sample standard deviation of the differences between predicted and actual values and NRMSE is useful to reflect the relative error between the modeled and measured soil moisture. The MBE was used to assess over or underestimation of VI-based ET_c_. The MAE evaluates the mean magnitude of the errors in predictions without considering their sign. The reason behind employing RMSE is its ability to measure accuracy to compare predicting errors of varying models for a particular dataset and not between datasets. The study used adjusted R^2^ because it provides a measure of how well the predicted values are replicated by the model based on the variability from the actual values. All the parameters are defined as follows: ${ETc}_{A}^{i}$ is the evapotranspiration based on theoretical K_c_ values, ${ETc}_{P}^{i}$ is the simulated evapotranspiration based on the spectral index derived and simulated K_c_ values $\;{ETc}^{-}$ is the mean value, *n* is the total number of data points and *k* is the number of variables in the model.

Adjusted R^2^:

(2) $$R_{adj}^{2}=1-\left[\frac{\left(1-\frac{\sum {{(ETc}_{p}^{i}-{ETc}_{A}^{i})}^{2}}{\sum {{(ETc}_{p}^{i}-{ETc}^{-})}^{2}})(n-1\right)}{n-k-1}\right]$$

Root Mean Square Error:

(3) $$RMSE=\sqrt{\frac{1}{N}\sum_{i=1}^{N}{{(ETc}_{p}^{i}-{ETc}_{A}^{i})}^{2}}$$

Normalized Root Mean Square Error:

(4) $$NRMSE=100\times \frac{\sqrt{\frac{1}{N}\sum_{i=1}^{N}{{(ETc}_{p}^{i}-{ETc}_{A}^{i})}^{2}}}{{ETc}^{-}}$$

Mean Bias Error:

(5) $$MBE=\frac{1}{n}\sum_{i=1}^{n}\left({ETc}_{p}^{i}-{ETc}_{A}^{i}\right)$$

Mean Absolute Error:

(6) $$MAE=\frac{1}{N}\sum_{i=1}^{N}\left|{(ETc}_{p}^{i}-{ETc}_{A}^{i})\right|$$

## 3. Results and Discussion

### 3.1. Trends of NDWI, NDRE, NDVI, and LAI Values for the Different Growth Stages of Maize

**Figure 2:**
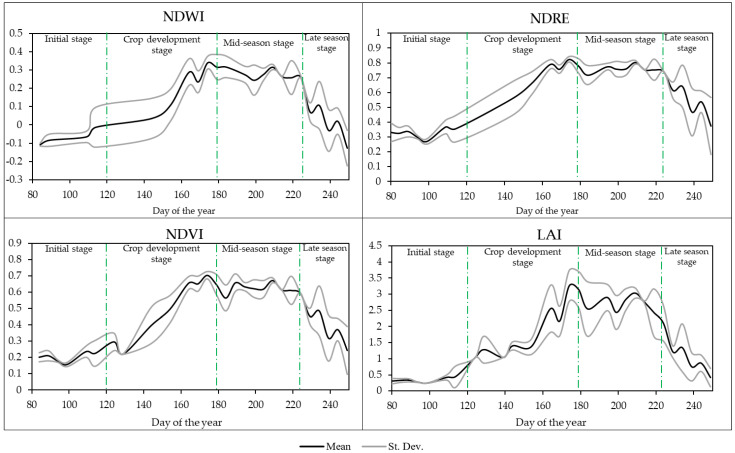
The mean NDWI, NDRE, NDVI, and LAI values over the three years (2018–2020).

The mean NDWI, NDRE, NDVI, and LAI values over the three years (2018–2020) for the different growth stages are presented in Figure 2. The NDWI is a commonly used index for monitoring vegetation water content. In the initial stage (days 80–140), the NDWI started with negative values, with a mean value of −0.05. Other authors shown that NDWI values for maize were low in the initial stage because the vegetation is still in its early stages of development and has not yet fully developed its leaf canopy [48,67]. As the plants grew and their leaf canopy expanded, the NDWI values began to increase steadily from 0.012 (day 120) in the crop development stage, reaching its maximum (0.374) on day 174 at the mid-season stage (days 141–224). These results are consistent with a study by Zhou et al. [68], which underscored the importance of vegetation monitoring by showing how effectively NDWI could capture the variability in canopy leaf abundance during specific phases. The amount of water stored in the plants decreases by a mean value of 0.0071 in the late season (days 225–249) due to the decreasing values.

The trends and distribution of NDRE values are similar to NDWI values. The mean NDRE values during the initial phase (days 80–140) were relatively low, at 0.2. This suggests that the leaves had little moisture content and that vegetation had not yet reached its full potential. In the crop development stage (days 141–179), the NDRE increased steadily as the vegetation grew, reaching a maximum on day 174 (0.82). During the mid-season (days 180–224), the mean NDRE values were similar to those around 0.76 at the crop development stage, similar to Liu et al. [69], who found, among other things, that the NDRE index has the highest correlation with canopy nitrogen concentration in summer maize. In the late-season stage (days 225–240), NDRE values showed a decreasing trend due to the gradual depletion of crop moisture.

In the initial phase (days 80–140), the mean NDVI values were low, with a mean value of 0.21. According to Lima et al. [70], NDVI values were low in the early part of the phenological cycle because of the higher radiation absorption in the near-infrared band during this period. In the crop development (days 141–179), NDVI values gradually increased and reached a maximum on day 174 (0.34). In the mid-season stage, values ranged from 0.56 to 0.67 until day 224 (late season). Reyes-González et al. [32] and Mebrie et al. [71] suggest that the crop coefficient varies constantly during the growing season and cannot remain constant throughout the mid-stage. In the late stage, the NDVI values showed a decreasing trend. Several studies have been conducted to investigate NDVI at different growth stages. Wang et al. [72] found similar values at different stages. The initial NDVI value of 0–0.2 increased with plant development up to 0.3–0.6, reaching a maximum of 0.7–0.8. Hatfield and Prueger [64] and Ji et al. [73] also divided the maize growing season into four growth phases and calculated phenological information metrics for each growth phase based on NDVI time series, while other authors have divided the growth phase into three, six, or even ten stages [74,75,76].

The trends and distribution of LAI values were similar to the previous indices. At the initial stage (days 80–140), the mean LAI values were low, around 0.5, as the vegetation was not fully developed, and the number of leaves was low. This statement aligns with the findings of Towers et al. [77], which supports the observation that at the initial stage, the mean LAI values were low due to underdeveloped vegetation and a low leaf count. In the crop development stage, the values increased as already described above and reached a maximum on day 174 (3.26). In the mid-season stage (days 180–224), the vegetation maintains a mean LAI value of around 2.7. The trend of data aligns with the findings of Zhang et al. [78] which supports the observation that LAI values follow an upward trend during the crop development stage and maintain a mean value during the mid-season stage in maize canopies. The LAI values also show a downward trend as leaf area decreases, like the other spectral indices. Similar trends in LAI values have been reported by several authors [79,80,81].

Overall, the trends and distributions of NDWI, NDRE, NDVI, and LAI values were similar, and the data followed well the vegetation development and environmental conditions.

### 3.2. Deriving VI-Based Crop Coefficient for the Vegetation Period of Maize

**Figure 3:**
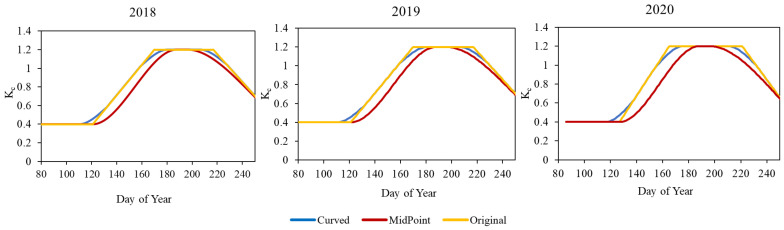
FAO-56-based (“original”, “MidPoint”, “curved”) K_c_ values for the years (2018–2020).

The lowest K_c_ values were observed during the initial stage (0.4) based on FAO-56. In the case of the “curved” dataset, it typically starts to gradually increase from this initial value about 10 days earlier compared to the “original” and “MidPoint” values. However, the “original” K_c_ values peak much earlier at the value of 1.2 in the crop development stage (days 166–170) than the other two datasets. Following the “original” K_c_ values, the “curved” dataset reaches its maximum (1.2) approximately 10 days later, followed by the “MidPoint” dataset after 7–10 days. As illustrated in Figure 3, this plateau phase represents approximately a 50-day period in the original K_c_ values, a 26-day period in the “curved” dataset for 2018 and 2019, and a 35-day period in 2020. In the case of the “MidPoint” dataset, this phase lasted for about only 8–9 days.

There was a significant correlation between FAO-56-based K_c_ values and VIs, thus proper regression models were capable of describing the real crop evapotranspiration based on the evaluated vegetation index in this study (Table 3). The correlation between Vis and FAO-56-based K_c_ smoothed by the “MidPoint” method was found to be the highest, therefore “MidPoint” K_c_ was selected to use for setting regression models between the VIs and FAO-56 K_c_. The correlation matrix indicates that LAI was less effective in reproducing the temporal pattern of the theoretical curves, while NDWI and NDVI seem to be the most effective in predicting Etc.

**Figure 4:**
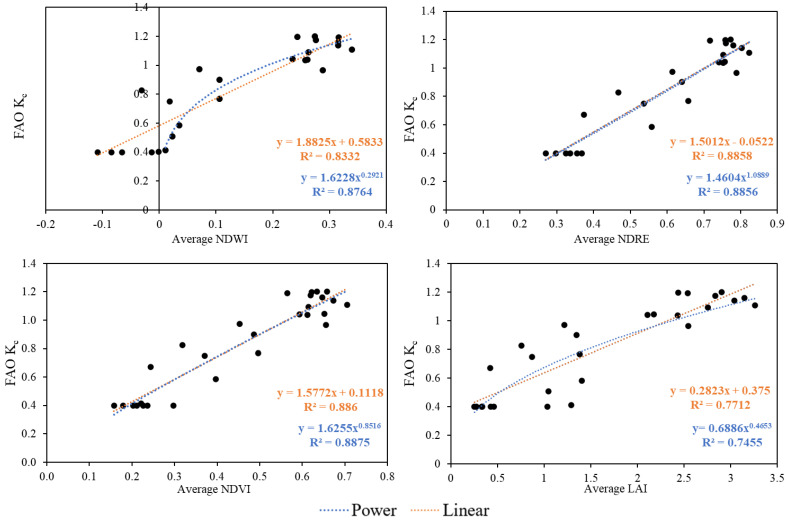
The linear and power regression models between the “MidPoint” K_c_ and the NDWI (*n* = 132), NDRE (*n* = 132), NDVI (*n* = 132), and LAI (*n* = 132) values of maize.

Both linear and power regression models were set based on “MidPoint” K_c_ and the mean NDWI, NDRE, NDVI, and LAI values (Figure 4). The linear correlation between mean NDWI values and FAO crop coefficient of maize (R^2^) was 0.83, indicating a strong positive correlation between the two variables. The R^2^ value of the power trend line was even higher (0.88). However, we could only fit this to the positive integers, which cover the period from day 129 to day 234 of the year, while the total growth stage was from day 84 to day 249. The relationship between NDRE and FAO K_c_ indicates a strong positive correlation for both linear and power fitting. The values were almost identical, R^2^ being 0.8858 in the former and 0.8856 in the latter case.

The correlation between NDVI and FAO data for maize has been investigated in several studies. In this study, for the NDVI, almost the same can be stated as for the NDRE index, as the R^2^ values were almost the same for both linear and power trend lines (R^2^ = 0.886 and 0.8875) and show a strong correlation between NDVI and FAO K_c_. The relationship between the NDVI and the crop coefficient (K_c_) for maize has been extensively studied. A strong linear association between the NDVI and FAO crop coefficient values was also discovered by Costa et al. [82]; the former’s value was 0.86, while the latter was 0.79. Reyes-González et al. [66] discovered that the NDVI and K_c_ FAO-56 had an even greater coefficient of determination of 0.97. Javed et al. [83] also reported that crop coefficients are highly correlated with satellite-derived NDVI values, suggesting a direct proportional relationship between the maize crop coefficient (FAO-56) and NDVI. Costa et al. [82] revealed a significant correlation between NDVI and the K_c_ values reported in the FAO-56 report with R^2^ = 0.794, an indication that there is a high correlation within these parameters. Singh and Irmak [31] formulated a linear regression model to determine the correlation between NDVI and ET_0_-based K_c_. The results showed that the coefficient of variation for both NDVI and K_c_ was lower for maize, soybeans, sorghum, and alfalfa at midseason compared to the early growing season as well as the late growth stage.

The lowest R^2^ was detected in the case of LAI between the index and the crop coefficient. The value for linear regression was 0.7712, whereas R^2^ in power fitting was slightly lower at 0.7455. According to Kang et al. [84], the K_c_ exhibited a rapid increase as the LAI approached 3 (linear R^2^ = 0.9522), maintaining elevated values when LAI exceeded 2.5 (linear R^2^ = 0.7612). The study by Jia and Wang [85] revealed a significant exponential relationship between LAI and crop coefficient K_c_ (R^2^ = 0.7055) under water deficit conditions, indicating the influence of vegetation indices on K_c_, and the impact of water stress on this relationship.

**Figure 5:**
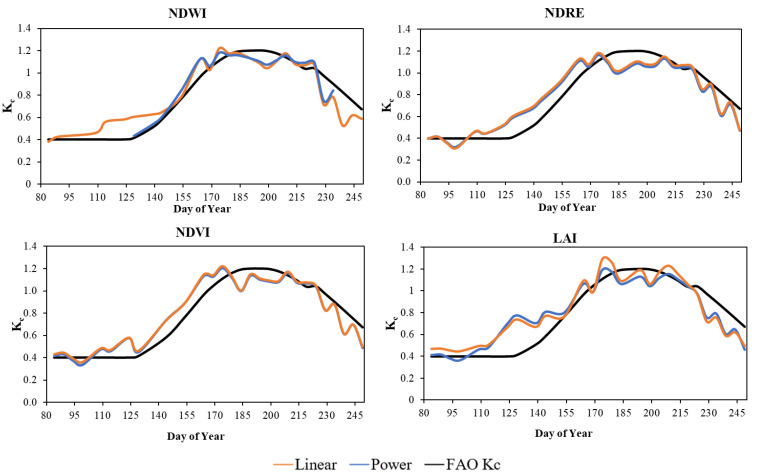
Comparison of FAO-56 crop coefficient, and linear and power-calibrated crop coefficient of maize with NDWI, NDRE, NDVI, and LAI indices.

Figure 5 illustrates the comparison between the FAO crop coefficient and crop coefficients estimated by linear and power functions for the growth stages. In the case of NDWI, in the initial stage (days 80–140), the linear calibrated K_c_ values were higher than the FAO K_c_ data, except on day 84, when the linear K_c_ was 0.379 and the FAO K_c_ was 0.4. The power-calibrated K_c_ data, as can be seen in Figure 5, could only be examined from day 129 onwards. In this short initial period (days 129–140), the power-calibrated K_c_ values and the FAO K_c_ values were almost the same. In the crop development stage (days 141–179), the power and linear calibrated K_c_ values were very similar (with a mean value of 0.99 in this stage) and had values higher than the FAO K_c_ (mean value of 0.94). In the mid-season stage (days 180–224), both FAO K_c_ and VI calibrated K_c_ values were above 1. Linear and power-calibrated values were again similar. The maximum crop coefficient value for the FAO data was on the 194th day (1.2), while for the power and linear calibrated data it was on the 174th day, 1.18 in the former case and 1.22 in the latter case. In the late season (days 225–250), values were again below 1, and similar to the mid-season, FAO K_c_ values were higher for both power and linear calibrated values. For the NDRE index, the linear and power-calibrated K_c_ values were almost equal in all stages. The FAO K_c_ data showed higher values than the calibrated data between days 91 and 115 of the initial stage (days 80–140), but for the remaining days of the initial stage and the whole crop development stage (days 141–179), the calibrated data were generally higher than the FAO K_c_ data. While in the initial stage, the mean of the calibrated data was 0.46 (for both power and linear), the FAO K_c_ was 0.41. The maximum values of power and linear calibrated K_c_’s were also the highest on the 174th day, 1.166 and 1.18, respectively. In the crop development stage, the mean of the calibrated K_c_ values for power and linear were 1.01 and 1.03, respectively, while the FAO K_c_ was 0.94. FAO K_c_ values were on average higher in the mid-season (days 180–224) and at the late season (days 225–250) stages, except for days 208–212 and 217–225, when linear calibrated K_c_ values were higher than both power calibrated and FAO K_c_ data. While the power and linear calibrated crop coefficient was 1.6–1.7 in mid-season and 0.7 in late season (for both power and linear). In the case of the crop coefficient values of the NDVI index, the linear and power-calibrated K_c_ values matched even better than in the case of the NDRE. The trend of the values was also similar to the trend already presented for the NDRE index, so the obtained K_c_ values from the power and linear fittings in the initial (days 80–140) and crop development (days 141–179) stages (0.44 and 0.45 in the initial stage, and 1.04 and 1.05 in the crop development stage) were higher than the FAO K_c_ mean values (0.4 in the initial stage and then 0.94 in the crop development stage). In the mid-season (days 180–224) and late-season stages (days 225–250), the FAO K_c_ values were higher (1.14 and then 0.82 with a mean value) than the power and linear calibrated K_c_ values. The maximum values of the power and linear calibrated K_c_’s for the NDVI were also highest on day 174, 1.207, and 1.224, respectively. For LAI, the calibrated values, on average the linear calibrated K_c_ values (mean value of 0.55), were higher in the initial phase (days 80–140). However, the highest value at this stage was produced by power-calibrated K_c_ values with 0.774 on day 129. This trend continued in the crop development phase (days 141–179), the mean value of the FAO K_c_’s was 0.94, while the calibrated K_c_ values exceeded 1. The maximum crop coefficient values for the power and linear calibrated data were 1.19 in the former case and 1.29 in the latter case. If the mean values were taken into account, the mean of the FAO K_c_ values were higher (1.13 and 0.82) in the mid-season (180–224th day) and late-season stages (225–250th day). Among the calibrated crop coefficients, in the mid-season, the linear calibrated values were higher (with a mean value of 1.12, while the power was 1.08), while in the mid-season the power calibrated K_c_ values were slightly higher (0.65—with a mean value of 0.64).

### 3.3. Estimated Crop Evapotranspiration of Maize along the Growth Stages

The estimation of crop evapotranspiration for maize at different growth stages is a critical aspect of agricultural water management (Table 4). The minimum NDWI-based crop evaporation was on day 80 for both the power (1.34 mm/day ± 0.102) and linear (1.37 mm/day ± 0.049) calibrated data, while the maximum value for power was on day 185 (6.45 mm/day ± 0.367) and for the linear calibrated ET_c_ data on day 170 (6.51 mm/day ± 0.434).

For NDRE, the minimum values were on day 95, which is still in the initial stage, with power at 1.36 mm/day ± 0.355 and linear calibrated ET_c_ at 1.29 mm/day ± 0.293. The maximum ET_c_ values were similar to the NDWI-based ET_c_, i.e., the power-calibrated ET_c_ had its highest value at mid-season (6.76 mm/day ± 0.337 on day 185), while the linear calibrated ET_c_ had its highest value towards the end of the crop development stage (6.4 ± 0.838 on day 170).

The evolution of NDVI-based K_c_ values was very similar to NDRE-based K_c_ values. The minimum value was on day 95, 1.31 mm/day ± 0.366 for the power calibration and 1.22 mm/day ± 0.286 for the linear calibration, and the maximum was on day 185 for the power calibrated K_c_ values (6.53 mm/day ± 0.331) and on day 170 for the linear calibrated values (6.46 mm/day ± 0.669).

The power-calibrated LAI-based K_c_ was lowest on day 80 (1.32 mm/day ± 0.015) and highest on day 185 (5.83 mm/day ± 0.492). In contrast, the linear calibrated K_c_ values had a minimum on day 95 (1.27 mm/day ± 0.293) and a maximum on day 170 (5.93 mm/day ± 0.322).

In general, the vegetation index-based crop coefficient values were lowest in the initial stage, while the highest values were towards the end of the crop development stage (for linear calibrated, etc.) and in the mid-season stage (for power-calibrated ET_c_).

### 3.4. Evaluation of Vegetation Indices (NDWI, NDRE, NDVI, and LAI) Based on ET_c_ Compared to FAO-56 K_c_

**Figure 6:**
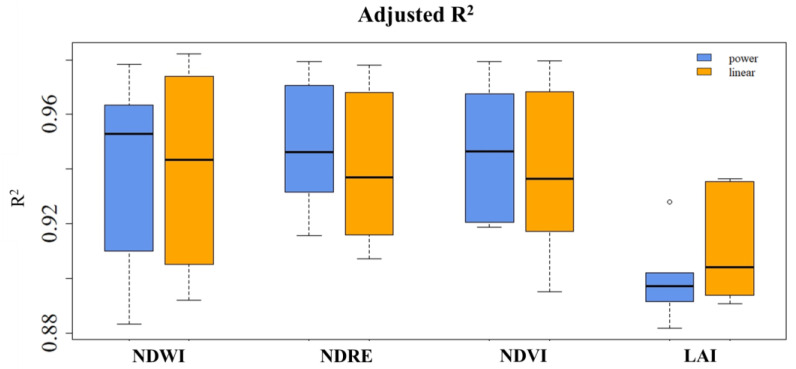
Adjusted R^2^ between ETc estimated with the vegetation indices (NDWI, NDRE, NDVI, and LAI) and that estimated with FAO-56 K_c_.

Based on the adjusted R-squared, the values closest to 1 were observed for the NDWI, NDRE, and NDVI indices, indicating that these models were able to explain a larger proportion of the variability in the data (Figure 6). For these three indices, the power regression model exceeded the linear regression model in general by a small margin. The LAI index had the lowest adjusted R-squared value, suggesting that the models could not fully account for the variability in the data. For LAI, the power regression model outperformed the linear regression model by a small margin; nonetheless, both models’ predictions displayed very significant variability, indicating that the models are less accurate in predicting LAI compared to NDWI, NDRE, or NDVI.

**Figure 7:**
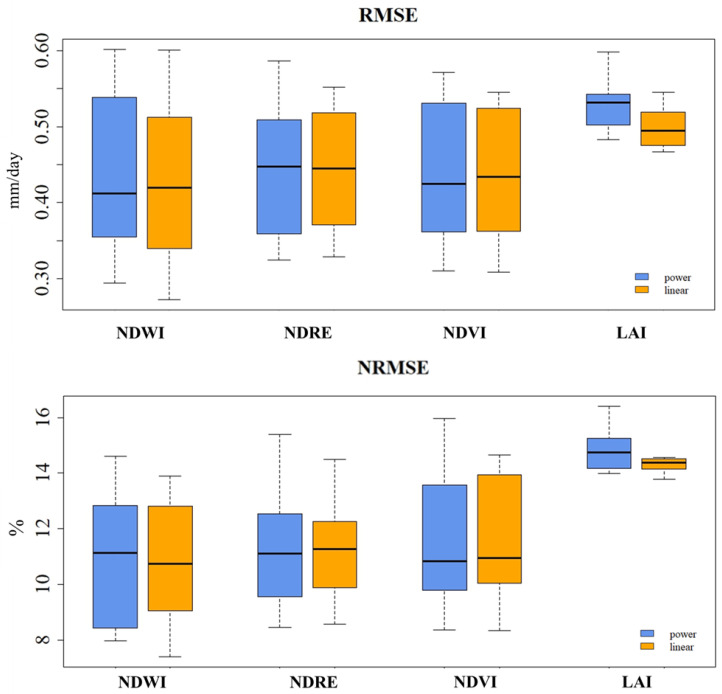
RMSE and NRMSE between ETc estimated with the vegetation indices (NDWI, NDRE, NDVI, and LAI) and that estimated with FAO-56 Kc.

According to the RMSE, the NDWI, NDRE, and NDVI indices exhibit similar RMSE values across both regression models (ranging between 0.43 and 0.45 mm/day), indicating comparable performance for predicting these values (Figure 7). The LAI index demonstrated the highest RMSE values for both regression models. However, the linear regression model (0.5 mm/day) appears to provide a slight advantage compared to the power regression model (0.53 mm/day).

The results for NRMSE were similar to those for the statistical indices described so far (Figure 7). For the NDRE index, the NRMSE values were relatively close, with the linear regression model achieving slightly lower values compared to the power regression model (11.36%). This indicates that both models perform similarly in predicting NDRE values. The values for the NDVI index also showed a mixed pattern. The linear regression model achieves relatively lower mean NRMSE values (11.56%) compared to the power regression model (11.48%), suggesting a small advantage for the linear regression model in predicting NDVI values. The LAI index exhibited the highest NRMSE values for both regression models.

**Figure 8:**
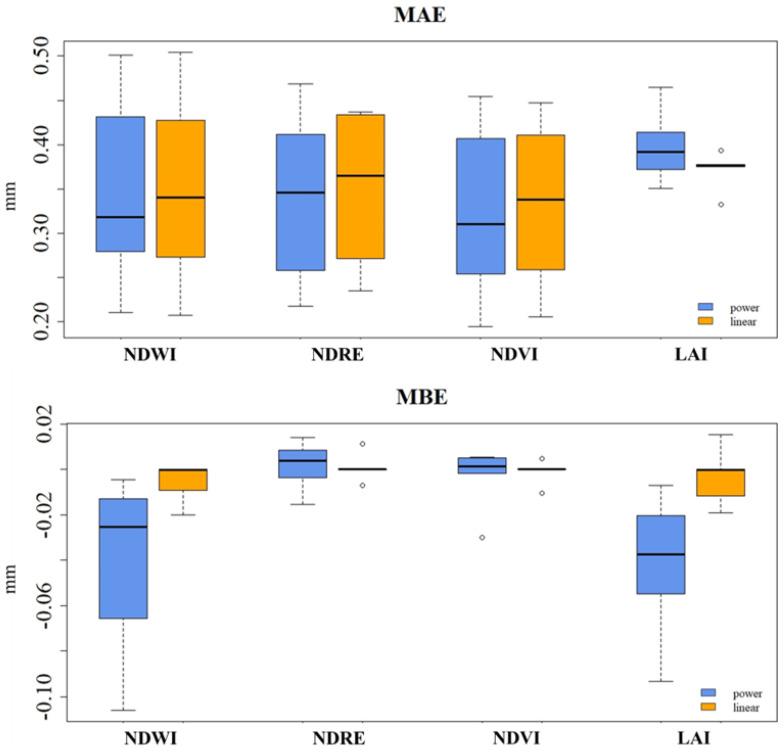
MAE and MBE between ETc estimated with the vegetation indices (NDWI, NDRE, NDVI, and LAI) and that estimated with FAO-56 Kc.

For MAE, the lowest values were observed for NDVI (0.32 and 0.33), but NDWI and NDRE values were also close to this (0.34 and 0.35) (Figure 8). The standard deviation of the values ranged from 0.08 to 0.11. Overall, the NDWI, NDRE, and NDVI indices exhibited similar MAE values across both regression models, indicating comparable performance for predicting these values. The LAI index demonstrates the highest MAE values, for both regression models, with the power regression model achieving a mean value of 0.40 mm/day and the linear regression model achieving a mean value of 0.37 mm/day.

In the case of MBE (Figure 8), the estimates of the linear regression model show mean smaller deviations from the actual values than the estimates of the power regression model. This was particularly the case for NDWI and LAI, where the mean NDWI for the power regression model was −0.04 mm and −0.005 mm for the linear model, while for LAI it was −0.042 mm and −0.003 mm. The NDRE and NDVI indices were closer to 0 for both the power and linear regression models.

## 4. Conclusions

In the Mediterranean, water competition among different stakeholders becomes more intense and affects agricultural production, especially in areas of high-intensive use like northern Italy. Changes in temperature patterns, rainfall, and extreme weather events affect agricultural outputs and make it difficult to plan optimal distribution of water and could lead to food insecurity. Effective water management becomes critical to addressing these challenges. For solving these issues, modern technological approaches such as RS provide effective solutions. In this study, researchers have created an innovative RS-based approach for calculating crop coefficients and evapotranspiration based on a case study site in Emilia Romagna, Italy. Through the use of remotely sensed indices coupled with climatic data, the accuracy in estimating crop evapotranspiration was substantially increased for maize fields.

The comparative study of NDVI, NDWI, NDRE, and LAI values in different stages of maize growth helps understand the changes that occur during vegetation development under changing environmental conditions. In addition, the relationship between these indices and K_c_ reveals their suitability in predicting crop evapotranspiration. The observed strong positive correlations between NDVI, NDWI, NDRE, and LAI values with FAO K_c_ indicate the possibility of using RS data to obtain reasonable estimates for crop water requirements. Regression models of NDVI, NDWI, and NDRE exhibit slightly different predictive performances in determining K_c_ values with power regression generally being superior to linear regressions. The most significant correlations are found for NDVI and NDWI, indicating their strength in predicting K_c_ values in maize. Nevertheless, there are lower correlation values for LAI which indicates that the predictive accuracy of this index is less than other indices.

Generally, NDWI, NDRE, and NDVI-based ETc estimations outperform consistently compared to LAI with power regression providing better results. In the end, it can be concluded that VI (NDWI, NDRE, NDVI, and LAI)-based crop evapotranspiration ETc are indicating different performances in predicting water requirements for crops compared to FAO-56 Kc-based ETc. NDWI, NDRE, and NDVI have good explanatory power with relatively accurate predictions for both models of regression while the LAI index shows some limitations in its accuracy. The power regression model usually slightly exceeds the linear regression model, NDWI, NDRE, and NDVI have better predictive accuracy than LAI. These results emphasize the importance of vegetation indices, especially NDWI and NDVI as promising approaches to estimate crop water requirements with further improvements required for LAI-based predictions.

In conclusion, the analysis shows that NDWI and NDVI indices are effective in monitoring maize growth stages and estimating crop evapotranspiration. Using RS data and regression modeling techniques provides appropriate tools for the improvement of agricultural water management practices to achieve higher crop productivity. More research and improvement of these methodologies can lead to more accurate and consistent strategies for crop water management in agricultural systems.

## Figures and Tables

**Table 1:** Summary of mean values for the soil parameters.

Parameter	Unit	Mean
Sand	%	32
Silt	%	50
Clay	%	18
pH	-	8.27
CaCO_3_ total	%	13.5
CaCO_3_ active	%	3.1
N total	%	0.06
K exchangeable	meq/100 g	0.34
P (Olsen)	meq/100 g	5.49
CEC	meq/100 g	21.6

**Table 2:** The lengths of the phenological stages.

Year	Stage	Period, T [Days]	K_c_ Range, [–]
2018	Initial	3 April 2018–28 May 2018	0.4–0.8
	Crop development	29 May 2018–29 June 2018	0.8–1.2
	Mid-season	30 June 2018–4 August 2018	1.2–0.9
	Late-season	21 July 2018–21 September 2018	0.9–0.6
2019	Initial	20 March 2019–20 May 2019	0.4–0.8
	Crop development	21 May 2019–8 June 2019	0.8–1.2
	Mid-season	9 June 2019–25 July 2019	1.2–0.9
	Late-season	14 July 2019–14 September 2019	0.9–0.6
2020	Initial	25 March 2020–11 May 2020	0.4–0.8
	Crop development	12 May 2020–1 June 2020	0.8–1.2
	Mid-season	2 June 2020–26 July 2020	1.2–0.9
	Late-season	13 July 2020–7 September 2020	0.9–0.6

**Table 3:** Correlation coefficients between estimated K_c_ curves.

			FAO-56		
		*n*	Original	Curved	MidPoint
Spectral indices	NDWI	132	0.941	0.914	0.952
	NDRE	132	0.934	0.888	0.939
	NDVI	132	0.942	0.901	0.952
	LAI	132	0.894	0.853	0.889

**Table 4:** Vegetation index (NDWI, NDRE, NDVI, LAI)-based estimation of crop evapotranspiration (ETc) (mm/day).

Day of Year		80	95	110	125	140	155	170	185	200	215	230	245
NDWI	power	1.34 ± 0.102	1.54 ± 0.534	2.01 ± 0.276	1.87 ± 0.242	2.46 ± 0.284	4.1 ± 0.646	5.88 ± 0.49	6.45 ± 0.367	6.32 ± 0.594	5.85 ± 0.545	4.7 ± 0.507	3.4 ± 0.704
	linear	1.37 ± 0.049	1.48 ± 0.428	1.97 ± 0.334	2.09 ± 0.408	2.6 ± 0.581	4.19 ± 0.958	6.51 ± 0.434	6.22 ± 0.572	6.12 ± 0.294	5.9 ± 0.453	4.54 ± 0.691	3.23 ± 1.229
NDRE	power	1.37 ± 0.041	1.36 ± 0.355	1.86 ± 0.1	1.72 ± 0.148	2.32 ± 0.221	4.13 ± 0.957	6.11 ± 0.557	6.76 ± 0.337	6.62 ± 0.34	6.11 ± 0.572	4.78 ± 0.223	3.31 ± 0.43
	linear	1.38 ± 0.07	1.29 ± 0.293	1.9 ± 0.302	1.89 ± 0.353	2.53 ± 0.365	4.54 ± 1.187	6.4 ± 0.838	5.92 ± 0.46	6.09 ± 0.248	5.93 ± 0.522	4.92 ± 0.409	3.23 ± 0.802
NDVI	power	1.32 ± 0.024	1.31 ± 0.366	1.78 ± 0.211	1.65 ± 0.249	2.23 ± 0.223	3.98 ± 0.941	5.89 ± 0.502	6.53 ± 0.331	6.4 ± 0.375	5.92 ± 0.538	4.57 ± 0.208	3.14 ± 0.431
	linear	1.36 ± 0.051	1.22 ± 0.286	1.82 ± 0.265	1.84 ± 0.364	2.42 ± 0.217	4.43 ± 1.162	6.46 ± 0.669	5.79 ± 0.72	5.92 ± 0.309	5.8 ± 0.438	4.64 ± 0.46	2.98 ± 0.735
LAI	power	1.32 ± 0.015	1.38 ± 0.332	1.83 ± 0.252	1.71 ± 0.323	2.22 ± 0.131	3.73 ± 0.912	5.31 ± 0.313	5.83 ± 0.492	5.74 ± 0.554	5.31 ± 0.45	4.17 ± 0.357	2.99 ± 0.302
	linear	1.34 ± 0.036	1.27 ± 0.293	1.87 ± 0.23	2.14 ± 0.283	2.26 ± 0.281	3.77 ± 1.427	5.93 ± 0.322	5.7 ± 0.618	5.92 ± 0.536	5.2 ± 0.686	3.66 ± 0.392	2.57 ± 0.368

## Data Availability

The original contributions presented in the study are included in the article, further inquiries can be directed to the corresponding authors.
